# Diffusion Tensor Imaging 3D Tractography-Guided, Individualized, Transsulcul Approach for Subcortical Hematoma Evacuation Using BrainPath/Myriad

**DOI:** 10.7759/cureus.81792

**Published:** 2025-04-06

**Authors:** Jose M Soto, Dongxia Feng, Yilu Zhang, Anthony Nguyen, Harold Sonnier, Jason H Huang

**Affiliations:** 1 Neurosurgery, Baylor Scott & White Medical Center - Temple, Temple, USA; 2 Radiology, Baylor Scott & White Medical Center - Temple, Temple, USA

**Keywords:** brainpath, corticospinal tract, dtt-guided, evacuation, myriad, subcortical hematoma, transsulcal

## Abstract

sICH (spontaneous intracerebral hemorrhage) is a major cause of death and disability. Traditional surgical evacuation, while beneficial, risks damaging healthy tissue. Minimally invasive surgery (MIS) offers a promising alternative. This study explores the feasibility and safety of diffusion tensor imaging (DTI)-guided, trans-sulcal MIS with BrainPath/Myriad NICO Corporation (Indianapolis, IN, USA) for sICH evacuation. DTI/tractography (DTT) visualizes critical pathways like the corticospinal tract (CST), aiding surgical planning and potentially predicting motor function recovery post-surgery.

Our objectives include i) assessing the feasibility and safety of DTT-guided, trans-sulcal MIS with BrainPath/Myriad for sICH evacuation. ii) Evaluating DTT's utility in surgical planning and its potential role in predicting motor function recovery.

Three sICH patients underwent pre-operative DTT with CST involvement graded A (direct injury) to E (displacement). Based on DTT, surgical trajectories using three trans-sulcal approaches were planned to avoid the CST. MIS with BrainPath/Myriad was performed aiming for <15 mL residual hematoma. Post-operative DTT and motor function follow-up (≥3 months) were conducted.

Three patients completed pre- and post-operative DTT scans. All were middle-aged males with sympathomimetic abuse history. Two had Type A CST involvement, and one had Type D. Both Type A patients recovered well but showed no significant motor improvement. The Type D patient showed motor improvement. All patients completed the three-month follow-up.

Our limited data suggests that DTI-guided, trans-sulcal MIS with BrainPath/Myriad for sICH evacuation is feasible and safe. DTT seems valuable for surgical planning and potentially predicts motor function recovery. Further studies with more patients are needed to confirm these findings.

## Introduction

Spontaneous intracerebral hemorrhage (sICH) is a source of significant morbidity and mortality. There are almost 80,000 cases of sICH per year in the US with an early mortality rate of 30-40%[1]. Growing evidence shows that motor outcome after stroke is heavily dependent on the integrity of the corticospinal tract (CST) [2]. Although large, superficial lesions (i.e., those within 1 cm of the cortical surface) can be readily evacuated to improve patient outcomes in certain circumstances, for subcortical lesions the current standard of care remains medical management [1]. Traditionally, subcortical lesions are approached via a large corticotomy using brain retractor blades that often cause significant white matter tract disruption. The concurrent cortical and subcortical trauma typically negates the potential benefits of hematoma removal. Multiple studies have shown that subcortical injuries are more severe than cortical injuries of the same volume [3-13]. Additionally, the inability to identify white matter tracts put them at risk of inadvertent injury.

New surgical techniques are being developed to improve outcomes in patients with subcortical lesions. One of these techniques utilizes the minimally invasive parafascicular approach to subcortical lesions [14,15]. Using stereotactic image guidance, a tubular retractor system is inserted through the cortex and subcortical tissue in a trans-sulcal manner. This approach reduces traction injury by the radial distribution of the retraction force and reduces parenchymal trauma by using a sulcus as a starting point rather than a gyrus. It also spares the projection fibers that originate from the gyri. The NICO Corporation (Indianapolis, IN, USA) has patented a system that facilitates this approach known as BrainPath. A recent study has shown improvement in functional outcomes using this device in patients with 30 to 80 milliliter (mL) hemorrhages [16]. Current national guidelines recommend considering evacuation of hemorrhages greater than 20-30 mL in patients with Glasgow Coma Scores (GCS) between 5 and 12 [1].

A novel adjunct to this technique is the use of diffuse tensor imaging 3D tractography (DTT) to identify subcortical white matter tracts so that they can be avoided during surgery. This tractography is derived from standard magnetic resonance imaging (MRI) sequences and processed using specialized software. Specifically, the major corticospinal tracts can be identified and avoided to reduce postoperative neurological deficits [7,15-20]. In this study, patients with subcortical intracerebral hemorrhages will undergo DTT to map out their corticospinal tracts prior to surgery. This information will guide the surgical approach to the clot. Additionally, DTT can be used postoperatively to evaluate the integrity of the CST to guide prognosis [21-23].

## Case presentation

Methods

This study was approved by our institutional review board. We enrolled three patients who fit our inclusion criteria for minimally invasive sICH evacuation. They underwent pre-operative DTT, and the involvement/injury of the CST was graded from A to E, with A signifying direct injury (i.e., a disrupted CST) and the others (B, C, D, and E) signifying displacement in anatomic directions (anterior, posterior, medial, and lateral, respectively). Grading was performed by the senior author (DF) in conjunction with another author (YZ). Surgical trajectories were planned from three trans-sulcal approaches (anterior, posterior, and lateral) to avoid damage to the ipsilateral CST, and the surgery was performed with the BrainPath and Myriad devices (NICO Corporation, Indianapolis, IN, USA). The anterior corridor was defined as the junction of the superior frontal sulcus and precentral sulcus and runs parallel to the association fibers. Its course runs between the superior longitudinal fasciculus and the cingulum. The parieto-occipital sulcus demarcated the posterior corridor, and the superior temporal sulcus was used as the lateral corridor. Additionally, fractional anisotropy (FA) and apparent diffusion coefficient (ADC) values were calculated for both bilateral CSTs before and after surgery. The posterior limb of the internal capsule was the region of interest for these calculations. DTT processing was performed by one of our authors (YZ), a neuroradiologist with specialized training in DTT generation. DTT data were analyzed using Food and Drug Administration-approved software (NordicBrainEx, v.2.3.9, NordicNeuroLab).

All surgeries were performed by the senior author (DF) in a consecutive fashion. Patients were positioned supine in 3-point fixation with a head clamp. Medtronic (Dublin, Ireland) Stealth navigation was used to mark out an appropriate corridor to the hematoma. A 3.5 cm craniotomy was used in all surgeries to place a BrainPath sheath, and the hematomas were evacuated using the Myriad device under microscope visualization. The goal of surgery was a residual hematoma volume of less than 15 mL, which is a common goal for evacuation in other trials [14]. After surgery, three patients underwent a second DTT imaging for assessment of their CSTs after surgery. All patients were followed clinically for improvement in their motor function. Demographic information was collected on all patients, and the patients have been followed clinically for at least three months.

Case 1

**Figure 1:**
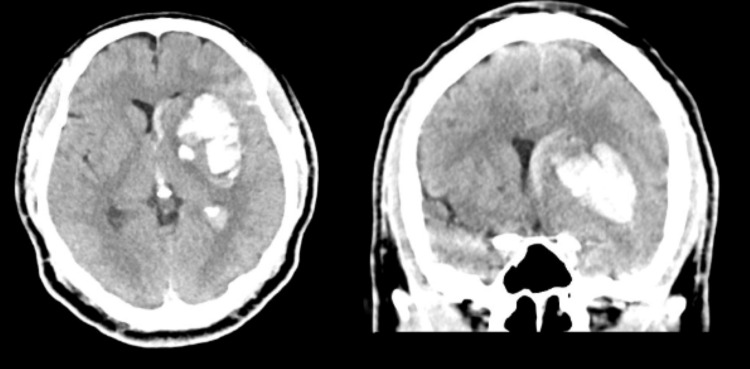
Preoperative CT scan for patient 1 CT scan with a left basal ganglia intracerebral hemorrhage (ICH)

**Figure 2:**
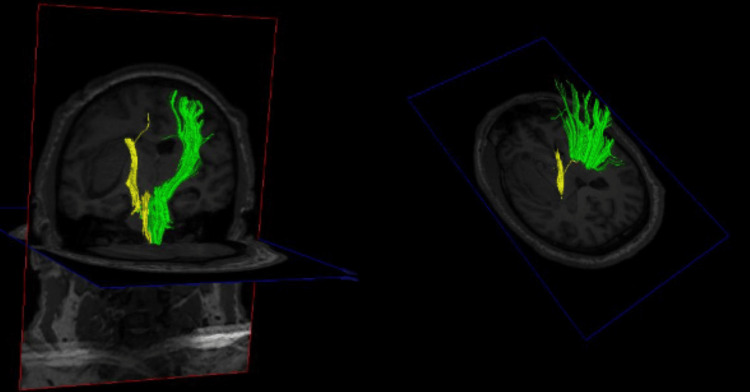
Preoperative DTT for patient 1 Preoperative DTT demonstrating injury to the left CST (yellow) DTT: Diffusion tensor imaging tractography, CST: Cortical spinal tract

**Figure 3:**
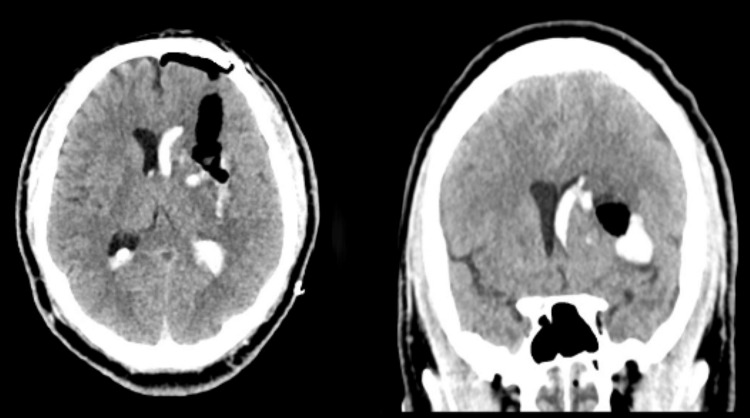
Post-operative CT scan for patient 1 CT scan with a left basal ganglia intracerebral hemorrhage (ICH)

**Figure 4:**
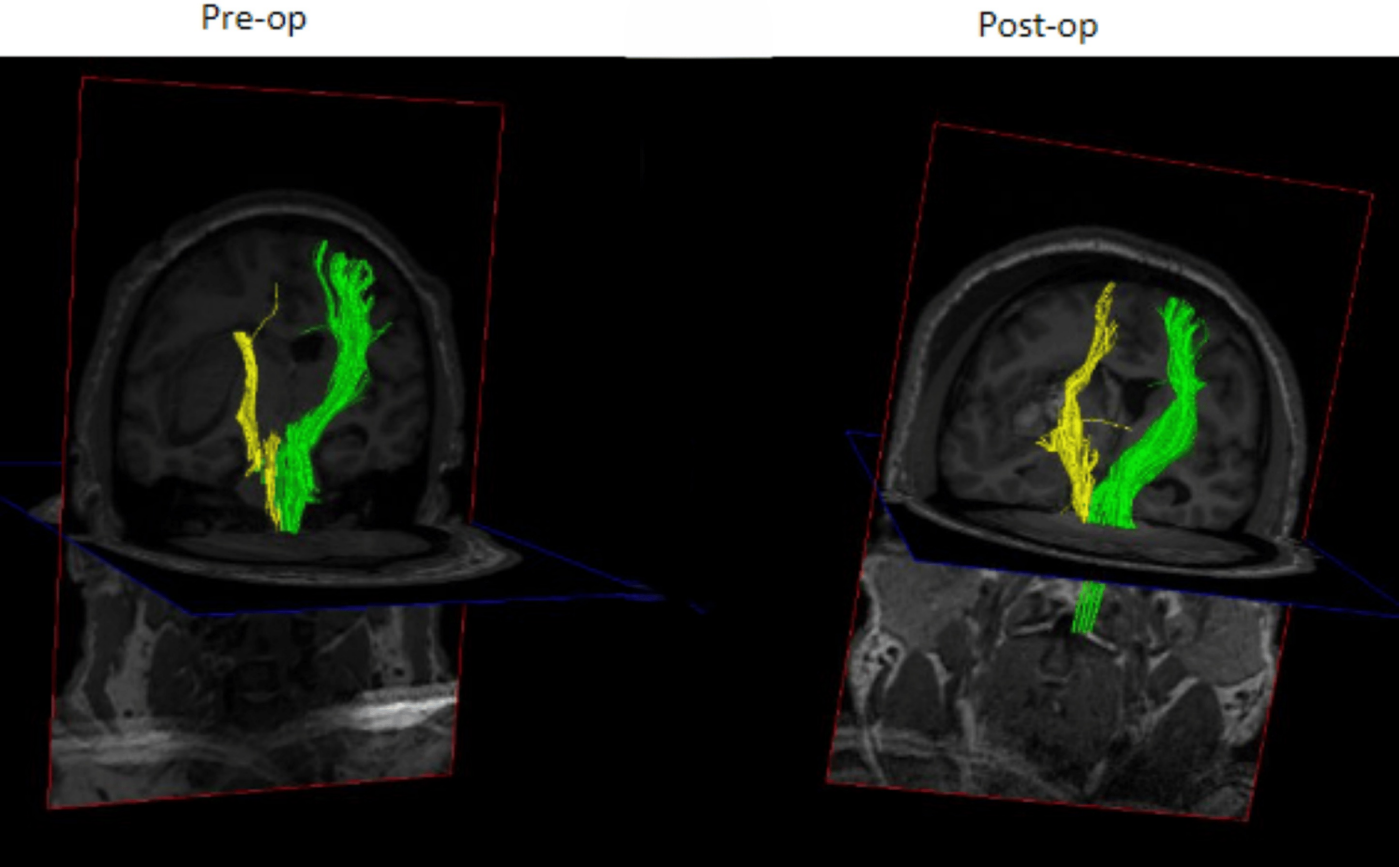
Pre- and post-operative DTT for patient 1 showing increased conspicuity of the ipsilateral CST Pre- and post-operative DTT demonstrating increased connectivity of the CST after evacuation of the ICH. DTT: DTI/tractography, CST: Cortical spinal tract, ICH: Intracerebral hemorrhage

A 41-year-old male with a history of methamphetamine abuse presented to the emergency department after he developed confusion and sudden right-sided weakness associated with right-sided facial droop. He had taken methamphetamine that evening when his symptoms started. He was intubated in the emergency department for airway protection. His exam was GCS 1/1T/5 with localization in his left upper/lower extremities and extensor posturing of his right-sided extremities. His initial computed tomography (CT) scan was consistent with a large left-sided subcortical sICH (about 45 mL in volume, Figure 1). Preoperative DTT demonstrated medical displacement of his CST (Grade D injury to the CST, Figure 2). A left frontal approach was selected, and the corridor and the hematoma were evacuated using a 75 mm BrainPath sheath. Postoperative CT was consistent with the satisfactory evacuation of the hematoma (Figure 3, residual hematoma volume of 6 mL). Additionally, repeat DTT after surgery was consistent with improved integrity of the ipsilateral CST (Figure 4). Both FA and ADC values increased after surgery, again consistent with improved integrity of the CST (Table 1). The patient recovered from his sICH well, and his last examination revealed almost full strength in his right lower extremity (strength grade 4/5) and some movement in his right arm (strength grade 1/5).

**Table 1:** Pre- and post-operative FA and ADC values for all three patients ADC: Apparent diffusion coefficient (in 10−3 mm^2^/s), CST: Cortical spinal tract, FA: Fractional anisotropy

Patient	Age (in years)/Sex	Pre-operative FA ipsilateralCST mean	Pre-operative ADC ipsilateral CST mean	Post-operative FA ipsilateral CST mean	Post-operative ADC ipsilateral CST mean	Pre-operative FA contralateral CST mean	Pre-operative ADC contralateral CST mean	Post-operative FA contralateral CST mean	Post-operative ADC contralateral CST mean
1	41/M	0.5435	73.31	0.5683	71.67	0.6109	71.95	0.5744	70
2	52/M	0.5927	64.39	0.5731	74.08	0.5494	71.89	0.5799	70.44
3	61/M	0.3984	85.82	0.3421	77.84	0.5918	74.03	0.4624	74.24

Case 2

**Figure 5:**
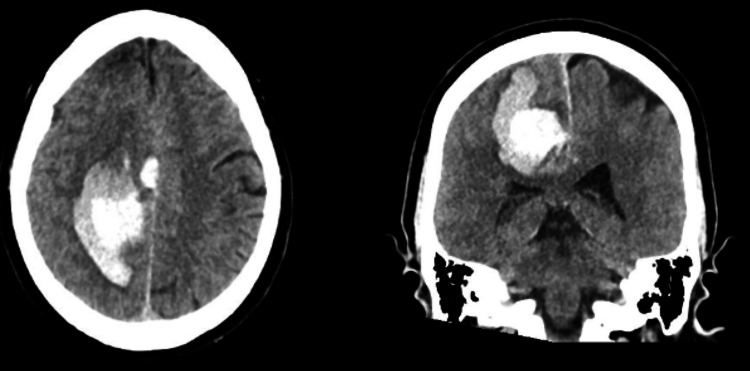
Preoperative CT scan for patient 2 Preoperative CT scan with a significant right frontal sICH sICH: Spontaneous intracerebral hemorrhage

**Figure 6:**
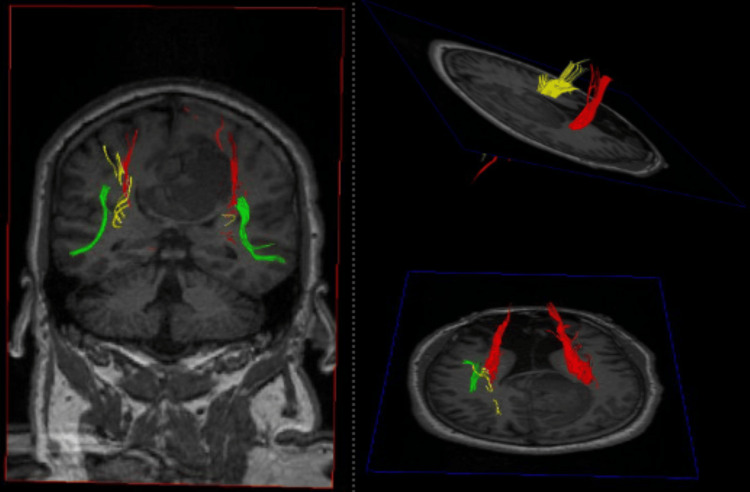
Preoperative DTT for patient 2 Preoperative DTT showing significant injury to the ipsilateral CST from the sICH DTT: DTI/tractography, CST: corticospinal tract, sICH: spontaneous intracerebral hemorrhage

**Figure 7:**
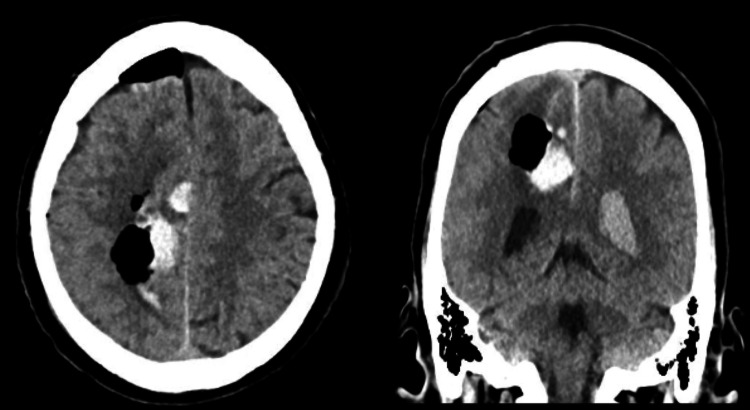
Post-operative CT scan for patient 2 Post-operative CT scan demonstrating excellent evacuation of the right frontal sICH sICH: Spontaneous intracerebral hemorrhage

**Figure 8:**
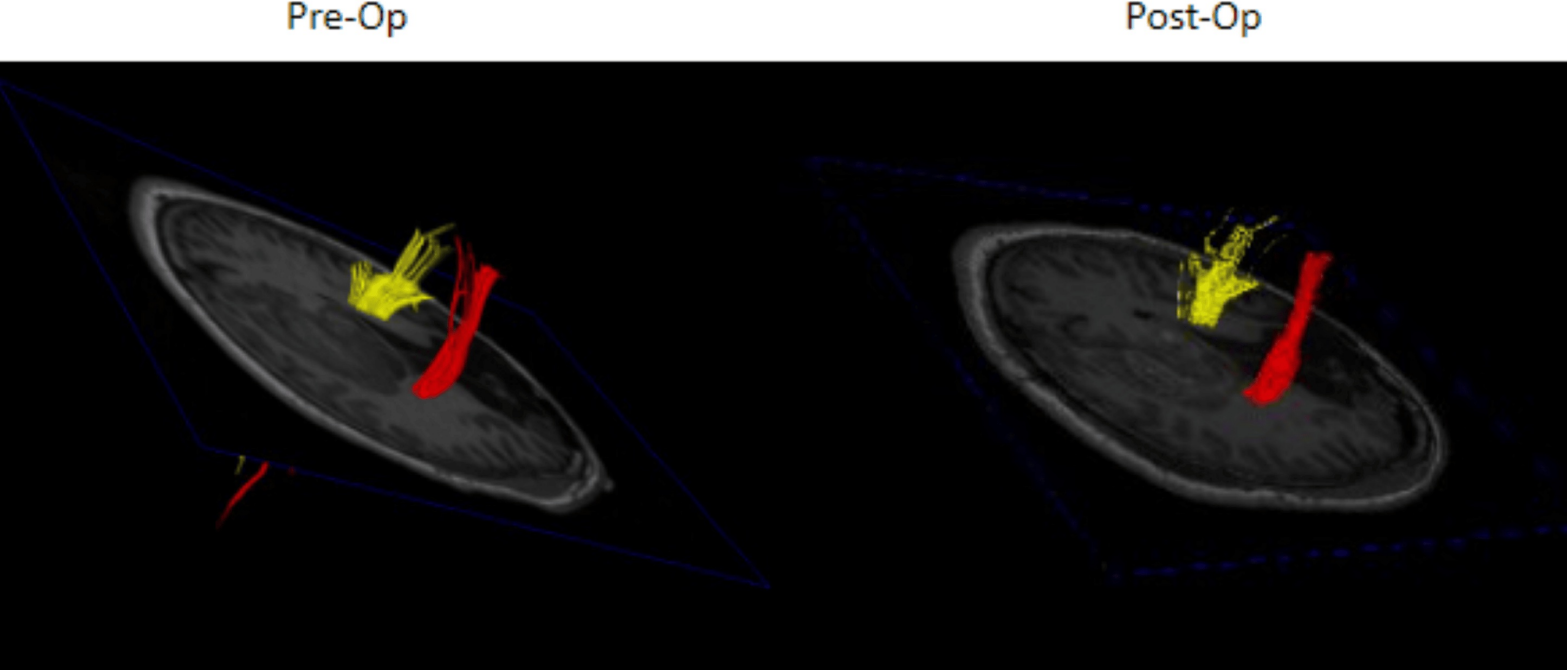
Pre- and post-operative DTT for patient 2 Comparison of pre- and post-operative DTT showing largely unchanged connectivity of the right-sided CST DTT: DTI/tractography, CST: Corticospinal tract

A 52-year-old male with a history of cocaine abuse presented to the emergency department with acute onset left arm and leg weakness. On his initial exam, he was GCS 4/4/6 with 1/5 strength in his left upper and lower extremities. His initial CT head demonstrated a large right frontal sICH (about 71 mL in volume, Figure 5). His pre-operative DTT was consistent with a Grade A (direct involvement) injury to the ipsilateral CST (Figure 6). The posterior corridor and a 60 mm BrainPath sheath were used to evacuate his hematoma because the remaining ipsilateral CST was displaced anteriorly and laterally. Post-operative CTH was consistent with a satisfactory evacuation (end of treatment volume of 11 mL), but post-operative DTT did not show improvement in the ipsilateral CST (Figures 7, 8). FA values decreased after surgery on the right side but increased on the left. ADC values increased on the right side but decreased on the left side (Table 1). He did well after surgery, but he did not regain voluntary movement of his left upper and lower extremities (he remained with 1/5 strength in those extremities).

Case 3

**Figure 9:**
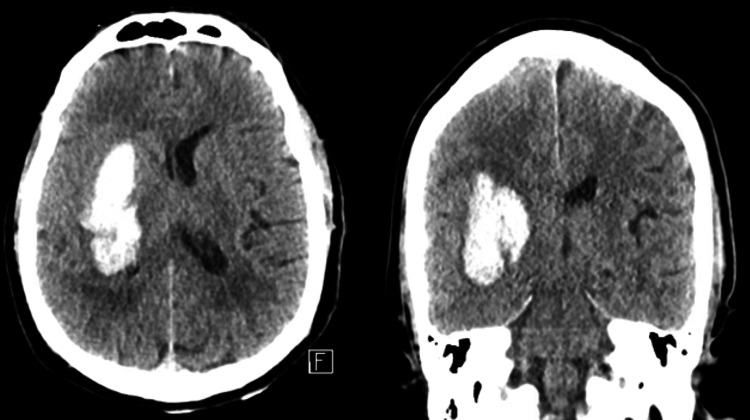
Pre-operative CT scan for patient 3 CT scan showing a large right basal ganglia ICH ICH: Intracerebral hemorrhage

**Figure 10:**
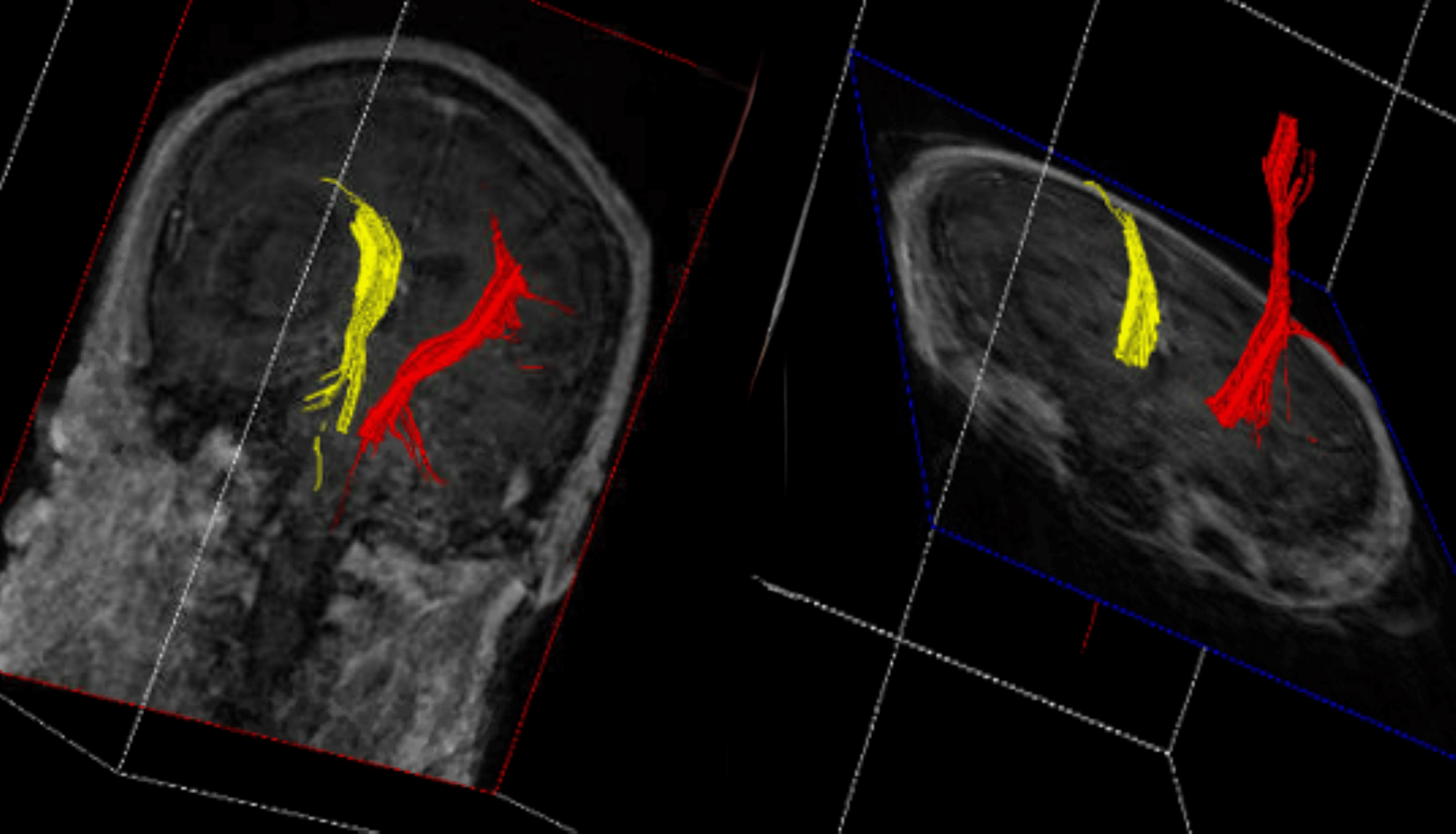
Pre-operative DTT for patient 3 Pre-operative DTT demonstrating injury to the ipsilateral CST DTT: DTI/tractography, CST: Corticospinal tract

**Figure 11:**
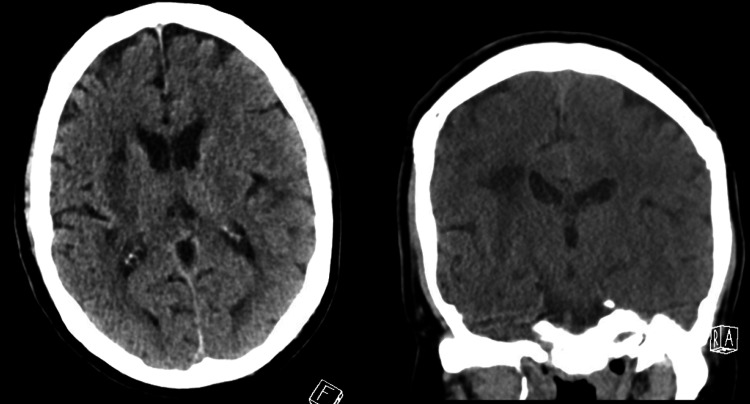
Post-operative CT scan for patient 3 Post-operative CT scan with complete evacuation of sICH sICH: Spontaneous intracerebral hemorrhage

**Figure 12:**
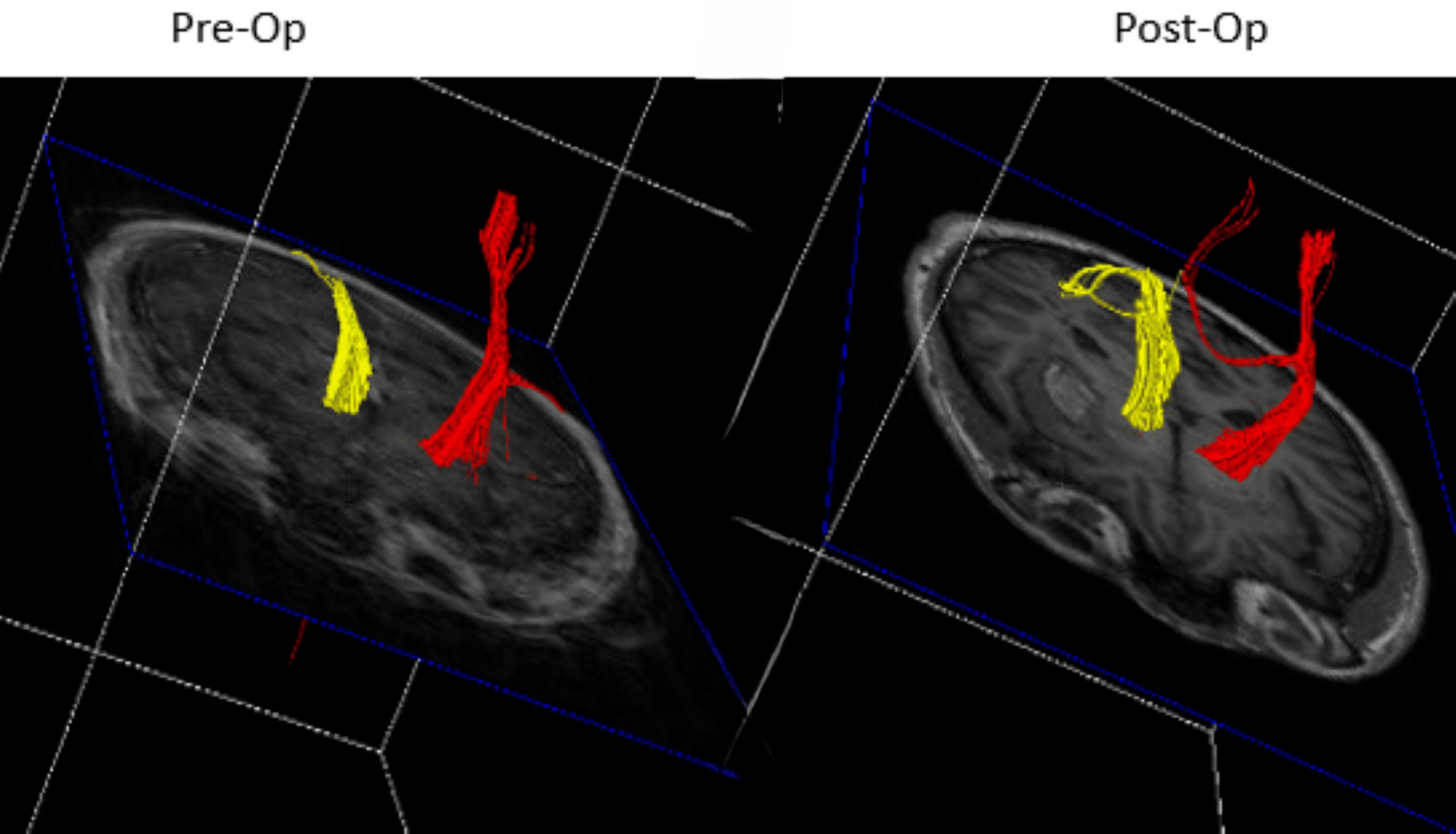
Pre- and post-operative DTT for patient 3 Comparison of pre- and post-operative DTT showing similar CST connectivity after evacuation of sICH DTT: DTI/tractography, CST: Corticospinal tract, sICH: spontaneous intracerebral hemorrhage

A 61-year-old male with a history of methamphetamine abuse presented to the emergency department with acute onset left arm and leg weakness. He was GCS 4/5/6 with 2/5 strength in his left arm and left leg. He was initially found to have a 15 mL right basal ganglia hemorrhage that expanded to 35 mL, and he became significantly weaker in his left hemibody (Figure 9). His mental status deteriorated, and his GCS became 2/3/6. His DTT was consistent with a Grade A injury (direct involvement, Figure 10). He was taken to the operating room for evacuation of his hematoma using the anterior corridor. A 75 mm BrainPath sheath was used in this case, and an end-of-treatment volume of 7 mL was achieved (Figure 11). His mental status improved after surgery, but he remained at 2/5 strength in his left-sided extremities. Post-operative DTT did not show significant improvement in the ipsilateral CST (Figure 12). Ipsilateral FA values also did not increase after surgery.

## Discussion

Our study demonstrates the feasibility and safety of DTI-guided, trans-sulcal minimally invasive surgery (MIS) using the BrainPath/Myriad system for spontaneous intracerebral hemorrhage (sICH) evacuation in a small cohort of patients. The integration of diffusion tensor imaging tractography (DTT) proved invaluable in surgical planning, allowing for the identification and avoidance of critical corticospinal tract (CST) pathways. This approach aligns with the growing recognition of the importance of preserving white matter integrity during sICH surgery, as evidenced by studies highlighting the detrimental impact of traditional open surgical techniques that often disrupt these pathways [11-13].

The ability of DTT to visualize the CST and classify the extent of injury (Grade A to E) facilitated the selection of optimal surgical corridors. In our case series, the anterior, posterior, and lateral trans-sulcal approaches were successfully employed, demonstrating the versatility of this technique. Notably, in Case 2, the posterior approach was crucial in avoiding further damage to the anteriorly and laterally displaced CST. This highlights the potential of DTT to tailor surgical strategies based on individual patient anatomy and pathology.

When considering surgical intervention for sICH, it's essential to contextualize our findings within the broader landscape of clinical trials. The STICH (surgical trial in intracerebral hemorrhage) and STICH II trials have significantly influenced the management of sICH [12,13]. These trials, while showing limited overall benefit from early surgery compared to conservative management, highlighted the complexity of sICH management and the importance of patient selection. Specifically, STICH II suggested potential benefits in patients with superficial lobar hemorrhages without intraventricular hemorrhage. However, both STICH trials relied on conventional surgical techniques, which often involve larger craniotomies and greater disruption of surrounding brain tissue.

Our study differs significantly in its utilization of minimally invasive techniques guided by DTT. We hypothesized that this approach, by minimizing tissue disruption and preserving critical white matter tracts, would improve outcomes compared to conventional surgery. The ENRICH trial, a more recent study evaluating MIS for sICH, demonstrated the benefits of lobar hemorrhages but met futility criteria for basal ganglia hemorrhages [16]. Crucially, the ENRICH trial, like the STICH trials, did not incorporate DTT for surgical planning. Our study, however, proactively utilized DTT to avoid the CST, which we hypothesized would improve outcomes. However, the limited data in our study, with patients who had direct CST involvement (Grade A) showing limited motor recovery, suggest that direct CST disruption may be a poor prognostic factor, regardless of surgical technique.

The changes observed in fractional anisotropy (FA) and apparent diffusion coefficient (ADC) values postoperatively provide insights into CST integrity. Increases in FA and decreases in ADC are typically associated with improved white matter organization and reduced edema, respectively. In case 1, the observed improvements in these metrics correlated with some degree of motor recovery. However, in cases 2 and 3, where direct CST injury was noted, the postoperative DTT and FA/ADC values did not show significant improvement, mirroring the lack of clinical motor recovery. This suggests that DTT may serve as a valuable tool for predicting motor outcomes postoperatively, allowing for more realistic patient and family counseling.

Several limitations must be acknowledged. First, the small sample size (n=3) restricts the generalizability of our findings and underscores the need for larger, multi-center studies. Second, the patient population, primarily middle-aged males with a history of sympathomimetic abuse, may not be representative of the broader sICH population, potentially introducing selection bias. Third, the absence of a control group limits our ability to definitively attribute the observed outcomes to the DTT-guided MIS technique. Future studies should include a control group (e.g., medical management, traditional surgery) to provide a more rigorous comparison. Fourth, while motor function was assessed, a more standardized and comprehensive assessment of functional outcomes using established scales such as the modified Rankin Scale or Barthel Index would enhance the study's rigor.

Despite these limitations, our study provides valuable preliminary evidence supporting the feasibility and potential benefits of DTT-guided, trans-sulcal MIS for sICH. Further research is warranted to validate these findings in larger, more diverse patient populations and to explore the long-term impact of this technique on functional recovery. Future studies should also explore the relationship between DTT metrics and functional outcomes in greater detail, potentially leading to the development of predictive models for personalized sICH management. Additionally, future trials should compare DTT-guided MIS directly against conservative management, and conventional surgical techniques, as used in the STICH trials, to understand if this new technique provides significant benefit.

## Conclusions

Based on our small sample size, using DTT in conjunction with MIS evacuation of sICH using the BrainPath/Myriad devices is feasible and safe. The information derived from DTT is useful both for surgical planning and may offer helpful prognostic information for recovery of motor function after sICH evacuation. We will use the data from this preliminary study to optimize our workflow and plan for larger studies involving more patients to see if using DTT-guided, trans-sulcal therapy will improve patient outcomes and lead to less post-operative motor deficits​.
